# Anti-tumor effects of Atractylenolide I on bladder cancer cells

**DOI:** 10.1186/s13046-016-0312-4

**Published:** 2016-03-01

**Authors:** Rui Yu, Bi-xia Yu, Jun-feng Chen, Xiu-yi Lv, Ze-jun Yan, Yue Cheng, Qi Ma

**Affiliations:** Department of Biochemistry and Molecular Biology, Zhejiang Key Laboratory of Pathophysiology, School of Medicine, Ningbo University, Fenghua St., 315211 Ningbo, China; Translational Research Laboratory for Urology, Ningbo First Hospital, The Affiliated Hospital of Ningbo University, Liuting St., 315010 Ningbo, China; Department of Urology, Ningbo First Hospital, The Affiliated Hospital of Ningbo University, Liuting St., 315010 Ningbo, China

**Keywords:** Atractylenolide I, Bladder carcinoma, Apoptosis, Cell cycle arrest, PI3K/Akt/mTOR

## Abstract

**Background:**

Atractylenolide I (ATR-1), an active component of *Rhizoma Atractylodis Macrocephalae*, possesses cytotoxicity against various carcinomas. However, little is known about the effects of ATR-1on bladder cancer. In the present study, the anti-tumor activity of ATR-1 was examined on bladder cancer cells both *in vivo* and *in vitro*.

**Methods:**

MTT assay was used to assess the cytotoxic effect of ATR-1. Cell cycle distribution and apoptosis levels were evaluated using flow cytometry. Western blotting assay was applied to measure the levels of proteins associated with the apoptotic pathway, cell cycle progression and PI3K/Akt/mTOR signaling pathway. Tumor models in nude mice were induced by injection of T-24 and 253J human bladder cancer cells.

**Results:**

ATR-1 inhibited bladder cancer cell proliferation, arrested cell cycle in G2/M phase through up-regulation of p21 and down-regulation of cyclin B1, CDK1 and Cdc25c. Meanwhile, ATR-1 also triggered cellular apoptosis depending on the activation of mitochondrial apoptotic pathway. Mechanism investigation indicated that ATR-1 exerts its anti-tumor effect also relies on the inhibition of PI3K/Akt/mTOR signaling pathway. Finally, mice studies showed that ATR-1 blocked the T-24 or 253J-induced xenograft tumor growth without noticeable toxicity.

**Conclusions:**

ATR-1 may be served as a potential therapeutic agent for the treatment of bladder cancer.

## Background

Bladder cancer is the second most common urological malignancies with an estimated 429,000 new cases and 165,000 deaths every year [1]. Most bladder cancer cases are superficial and treated with transurethral resection in the clinic. However, approximately 20 to 25 % of primary bladder cancers have invaded the muscle layer of the bladder wall at first diagnosed and radical cystectomy is needed [2]. Current treatments to bladder cancer have high recurrence rates and may cause strong side effects [3]. Although great efforts have been made in the treatment of bladder cancer over past decades, it still remains as a major health concern and new therapeutic approaches are urgently required [4].

**Fig. 1:**
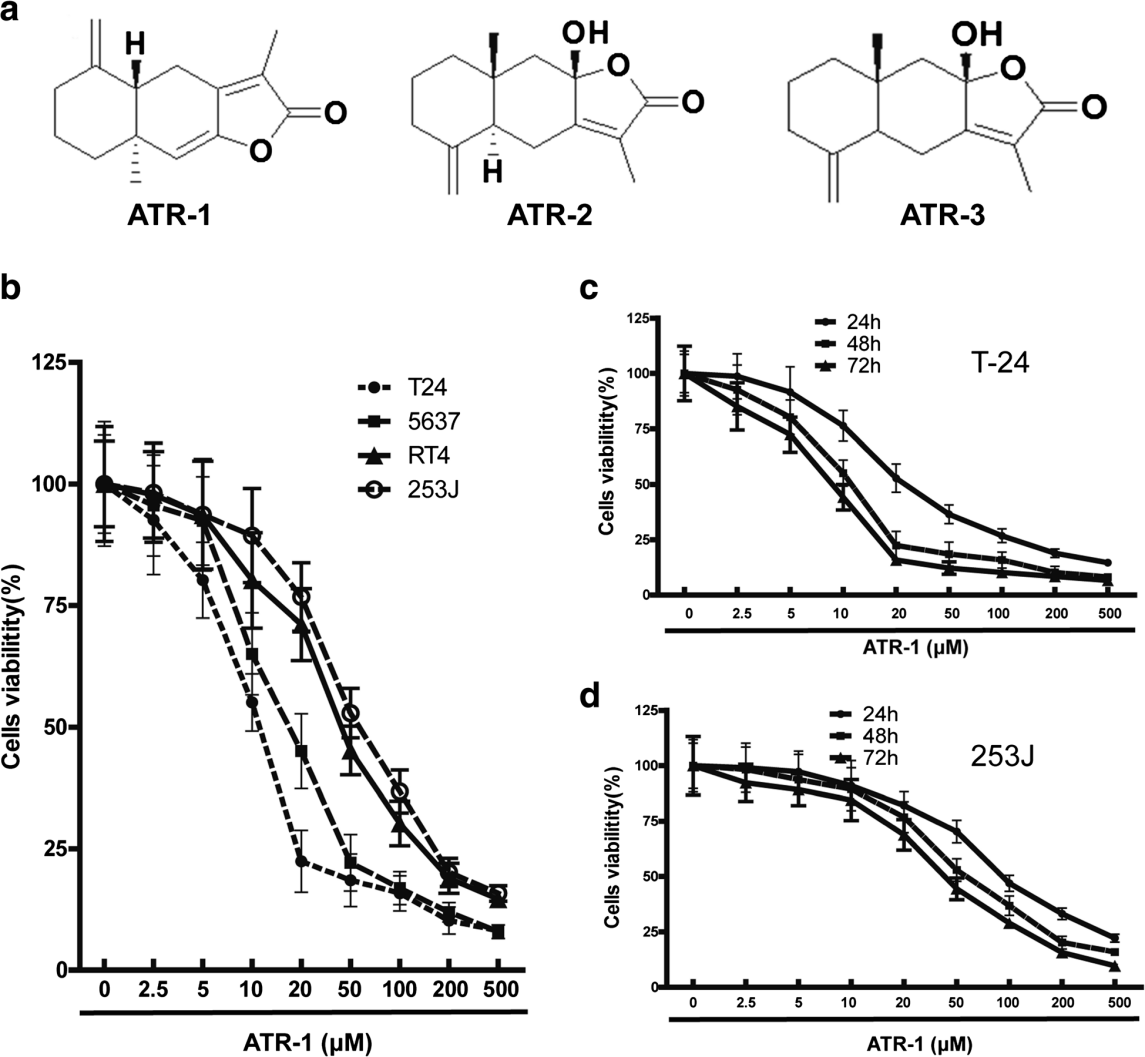
Effects of ATR-1 on the viability of bladder cancer cells. **a** Chemical structure of ATR-1, ATR-2 and ATR-3. **b** Bladder cancer cell lines RT4, 5637, T-24 and 253J, were treated with ATR-1 with different concentrations for 48 h. **c** T-24 and **d** 253J cells were treated by various concentrations of ATR-1 for 24 h, 48 h and 72 h. Cell viability determined by MTT assay as described in Methods section. Data represent mean ± S.D. (*n* =3)

In recent years, natural products have attracted great attentions for identifying new anti-tumor agents due to their high efficacy and low toxicity. Atractylenolide (ATR) can be extracted from *Rhizoma Atractylodis Macrocephalae*(RAM) which was used as an herb medicine in eastern Asia for a long history. ATR have three members named ATR-1, ATR-2 and ATR-3 respectively (Fig.1a). ATR exhibits a wide range of biological and pharmacological activities, such as anti-inflammatory, neuroprotection and gastroprotective effects [5–7]. Among the three members of ATR, ATR-1 exerts the best anti-tumor activity [8, 9]. For example, ATR-1 was able to trigger apoptosis and cell cycle arrest in melanoma cells as well as lung carcinoma cells *in vitro* [9, 10]. Meanwhile, ATR-1 could also suppress the growth of lung cancer *in vivo* [11]. Notably, a clinical study showed that ATR-1 treatment on gastric cancer cachexia patients can improve appetite and KPS (Karnofsky performance status) with few side effects [12]. These studies indicate that ATR-1is a promising and safe anti-tumor agent. However, the effects of ATR-1 on bladder cancer have never been reported and the mechanisms behind the anti-tumor activity of ATR-1 still need further investigation.

Cancer therapeutics often suppress cancers through interfering with cell cycle and/or triggering cellular apoptosis. The cell cycle progression is driven by cyclin-dependent kinases (CDKs) and cyclins, which act together to regulate cell cycle transition from G1 to S phase and/or from G2 to M phase [13]. Cell cycle deregulation leading to uncontrolled cell proliferation is one of the most common alterations during cancer development [13]. Therefore, blockage of cell cycle is considered as an effective cancer therapeutic strategy. Besides the cell cycle progression, apoptosis process is another vital target of anti-cancer agents. In eukaryotic cells, apoptosis can be triggered by two major signaling pathways: the extrinsic pathway and the intrinsic pathway which is also known as the mitochondrial pathway. The mitochondrial pathway is initiated with mitochondrial membrane potential (MMP) loss, releases of cytochrome c, lead to the activation of caspases, and ultimately resulting in chromatin condensation, DNA fragmentation and the formation of apoptotic bodies [14]. The mitochondrial pathway is regulated mainly by the Bcl-2 family members. Bcl-2 family members can be divided into two groups: pro-apoptotic members like Bax, Bad, and anti-apoptotic members like Bcl-2, Bcl-xl, Mcl-1 etc. Cancer cells often evade apoptosis due to the down-regulation of pro-apoptotic Bcl-2 proteins and/or up-regulation of anti-apoptotic Bcl-2 proteins. Therefore, agents with inhibitory activities toward anti-apoptotic Bcl-2 members may be valuable to fight against cancers.

Plenty of studies have confirmed that PI3K/Akt/mTOR signaling pathway is often constitutively activated and plays an important role in the development of various cancer types and resistance to anticancer therapies [15, 16]. Particularly, the PI3K/Akt/mTOR pathway was also found to be associated with a substantial number of bladder cancers. Amplification and/or mutations of several key genes regulating this pathway, such as *PIK3CA* (encoding the p110α subunit), *PIK3R1*(encoding the p85α subunit), *AKT*, and *PTEN*, are well-known mechanisms involved in the activation of PI3K/Akt/mTOR pathway in bladder cancer [17]. Moreover, inhibitors of the PI3K/Akt/mTOR pathway showed promising results in preclinical studies. For example, an inhibitor named RAD001 or everolimus could significantly inhibit the growth of bladder tumor both *in vitro* and *in vivo* [18]. Therefore, targeting PI3K/Akt/mTOR might be an effective strategy in the treatment of bladder cancer.

In the present study we investigated the anti-tumor activity of ATR-1on human bladder cancer cells and explored the underlying molecule mechanisms. We found ATR-1 inhibited bladder cancer cell proliferation, arrested cell cycle in G2/M phase, induced apoptosis through the mitochondrial pathway, and blocked the *PI3K/Akt/mTOR* pathway. Furthermore, ATR-1 suppressed xenograft bladder cancer growth *in vivo*. Thus, ATR-1 is a valuable natural product that may play a role in clinical usage for the treatment of bladder cancer.

## Methods

### Reagents and antibodies

ATR-1, Z-DEVD-fmk (caspase-3 inhibitor), Z-LEHD-fmk (caspase-9 inhibitor), 3-(4,5-dimethylthiazol-2-yl)-2,5-diphenyltetrazoliumbromide (MTT) and all other chemicals were purchased from Sigma-Aldrich (St. Louis, MO, USA). Antibodies against AKT, p-AKT, and PI3K were from Abcam Inc. (Cambridge, UK). Antibodies against CDK1, cyclin B1, Bax (6A7), mTOR, PTEN and p21 were purchased from Santa Cruz Biotechnology (Santa Cruz, CA, USA). Antibodies against caspase-9, caspase-3, Bcl-2, Mcl-1, Bcl-xl, Bad, Bax, Bak, Cytochrome-c, Smac/Diablo, Cox-IV, cdc25c, p-Cdc25cand β-actin were from Cellular Signaling Technology (Danvers, MA, USA).

### Cell culture and transfection

The bladder cancer cell lines were obtained from the Cell Resource Center, Shanghai Institute of Life Sciences, Chinese Academy of Sciences (Shanghai, China). The cell lines were cultured in RPMI 1640 media supplemented with 10 % fetal bovine serum, penicillin (100 U/mL) and streptomycin (100 μg/mL) on 37 °C with 5 % CO_2._ RPMI-1640 medium and fetal bovine serum (FBS) were purchased from Thermo Scientific (Rockford, USA). Penicillin and streptomycin were purchased from Gibco BRL, Invitrogen (Carsbad, CA, USA). All cells were split every 3 days and seeded at 1 × 10^5^ cells/ml onto plate 24 h before each experiment. For transfection studies, cells were transiently transfected with myr-AKT1 plasmid (constitutive active mutant) or the EGFP (control) plasmid with Fugene HD (Roche, Switzerland). The myr-AKT1 (#20422) plasmid was obtained from Addgene Company and EGFP plasmid was a generous gift from Dr. Hui Liu (Bengbu Medical College, Anhui, China). Bax siRNA or scramble siRNA were synthesized from Life Technologies (Shanghai, China) and transfected into cells using Lipofectamine 2000(Life Technologies, Shanghai, China) according to the manufacturer’s protocol. The sequence of Bax siRNA beginning at nucleotide 187 of the Bax gene, 5-′CTCCGGCGAATTGGAGATGAA-3′; Total cell lysates were harvested 48 h after transfection to assess the knockdown efficiency by western blot analysis.

### MTT assay

We used the MTT assay with minor modifications to measure cytotoxic activity of ATR-1 to different cells lines of bladder cancer cells [19]. The cells were seeded in 96-well plates (Corning, NY) with a density of 2 × 10^4^/well. When reaching 75–80 % confluence, the cells were exposed to DMSO (0.1 %) or different concentrations of ATR-1, which was dissolved in 0.1 % DMSO. Two days later, 25 μl MTT (Sigma, St. Louis, MO, USA) was added to the cultured media, and was removed at 4 h before the end of study. Finally, another 150 μl DMSO was added to each well, and the absorbance was measured by ELx800 ELISA Microwell Reader (BioTek Co, USA). The inhibitory IC50 values of ATR-1were calculated from the dose–response curves fitted by GraphPad Prism software (GraphPad Prism 5.01, GraphPad Software, Inc., CA, USA). The results were represented by mean values of three independent experiments.

### Apoptosis analysis

To detect apoptosis, cells were incubated with DMSO (0.1 %) or ATR-1 at different concentrations for 24 h. The cells were harvested, washed twice with cold 1 × PBS. The cells were then stained with FITC Annexin V Apoptosis Detection Kit (BD Biosciences) according to the manufacturer’s instruction and subjected to analysis by flow cytometry (FACScan, Becton-Dickinson, NJ, USA). The apoptosis was evaluated based on the percentage of cells with Annexin V+/PI+. The results were indicated as mean values from three independent determinations. The results were analyzed by Flowjo software. Five independent tests with the same conditions were performed.

### Western blotting analysis and immunoprecipitation

Western blot analysis was performed to measure the expression levels of various proteins in cells. Cells were treated with different concentrations of ATR-1 for 24 h. Cells were harvested, washed with cold 1 × PBS, and lysed with CHAPS lysis buffer (Cellular Signaling Technology, USA) for 30 min on ice, then centrifuged at 12,000 g for 15 min at 4 °C.The protein concentrations were measured by BCA protein assay kits (Sigma Aldrich, Saint Louis, Missouri, USA). Equal quantities of protein were loaded and separated by 12 % SDS-PAGE, and then transferred to a PVDF membrane (Millipore Inc. MA, USA). The blots were blocked in 12 % non-fat milk or 5 % BSA, and incubated with primary antibodies, followed by incubation with secondary antibodies conjugated with horseradish peroxidase (HRP). Ultimately, the protein bands were visualized by enhanced chemiluminescent reagents (Thermo scientific, Rockford, USA). For Bax immunoprecipitation, equal amounts of protein from whole cell lysates (800 μg) were used for immunoprecipitation. All samples were brought to a final volume of 450 μl with CHAPS lysis buffer (1 % CHAPS, 10 mM Hepes pH 7.4, 150 mM NaCl, and protease inhibitors). Samples were then rotated for 5 h at 4 °C with 5 μl of monoclonal antibodies (Bax 6A7, 200 μg/ml) and 150 μl anti-rabbit IgG magnetic beads. Then beads were precipitated by a magnetic field and washed five times with CHAPS buffer. Finally, the last supernatant was removed and 25 μl of 5 × loading buffer added. The beads were incubated in the loading buffer at 95–100 °C for 5 min, and then centrifuged at 12,000 rpm for 5 min. The supernatants were subjected to Western blotting analysis.

### Preparation of subcellular fractions

In order to separate the cytosolic and mitochondria fractions, cells were washed in ice-cold PBS. The cells were then lysed using CLAMI (Cell Lysis and Mitochondria Intact) buffer (250 mM Sucrose, 80 mM KCl, 50 μg/ml Digitonin in PBS) on ice for 5 min, and the cell suspension was centrifuged at 1500 rpm for 5 min at 4 °C. The supernatant was removed and stored at−20 °C as the cytosolic fraction.

### Cell cycle analysis

Cell cycle status was detected by flow cytometry and analyzed by Flowjo software. Briefly, cells were seeded onto 6-well plates at a density of 1 × 10^6^ cells/well and incubated for one day. After treatment with various concentrations of ATR-1 for 24 h, the cells were then harvested and washed with 1× PBS. Cell pellets were fixed in 70 % cold ethanol overnight. The fixed cells were resuspended in 1 × PBS containing 1 mg/ml RNase A (Sigma-Aldrich, USA), incubated for 1 h at 37 °C, and the cells were stained by adding 50 μg/ml PI (Sigma-Aldrich, USA) for 30 min at room temperature in the dark. The cells were then analyzed using flow cytometry (FACScan, Becton-Dickinson, NJ, USA). The results were indicated as mean values from three independent determinations.

### Tumor xenograft model

The animal study was approved by Ethical Committee on Animal Research of Ningbo University. Four week old male BALB/c nude mice were purchased from the Shanghai Laboratory Animal Center, Shanghai Institute for Life Sciences, Chinese Academy of Sciences (Shanghai, China). To induce tumors in nude mice, cell lines were harvested and suspended in RPMI-1640 medium. Then the tumor was prepared by injection of 5 × 10^6^ cells at right area of nude mice. When the size of tumors reached to 6–9 mm in diameter, the tumor-bearing mice were randomly divided into control and treated groups (8–9 mice for each group), which received intraperitoneal administration of DMSO (0.1 %) or ATR-1(25, 50, 75 mg/kg/day) for 4 weeks. Meanwhile, the sizes of tumor were periodically measured by calipers, and the volume was obtained by following formula: tumor volume (mm^3^) = maximal length (mm) × (perpendicular width) (mm)^2^/2. At the end of study, the mice were killed, and the tumors were removed for further measurement of weight.

### Statistical analysis

SPSS software (version 12; SPSS Inc., Chicago, IL, USA) was used for statistical analysis. Data are presented as the mean ± the standard deviation. Differences between groups were analyzed using the one-way ANOVA and Student’s *t*-test. A difference was considered significant at *P* < 0.05.

## Results

### ATR-1 suppresses proliferation of bladder cancer cells lines

The anti-proliferative activity of ATR-1 was initially evaluated by using four human bladder cancer cell lines RT4, 5637, 253J and T-24. Cells were exposed to various concentrations of ATR-1 for 48 h, and the cell viabilities were measured by MTT assay. As shown in Fig. 1b, ATR-1 inhibited the proliferation of RT4, 5637, 253J and T-24 in a dose-dependent manner. Accordingly, IC50 values of ATR-1 were determined as 44.5, 18.4, 63.7 and 12.8 μM, respectively (Table 1). We noticed that the proliferation of T-24 and 253J were also reduced by ATR-1 treatment in a time-dependent pattern (Fig. 1c, d).

**Table 1:** IC50 values of bladder cancer cell lines treated with ATR-1 for 48 h

Cell lines	IC50 values (μM)
T-24	12.8 ± 2.6
5637	18.4 ± 3.1
RT4	44.5 ± 5.3
253J	63.7 ± 7.4

Data represent mean ± S.D. (*n* = 3)

### ATR-1 induces cell cycle arrest at the G2/M phase

**Fig. 2:**
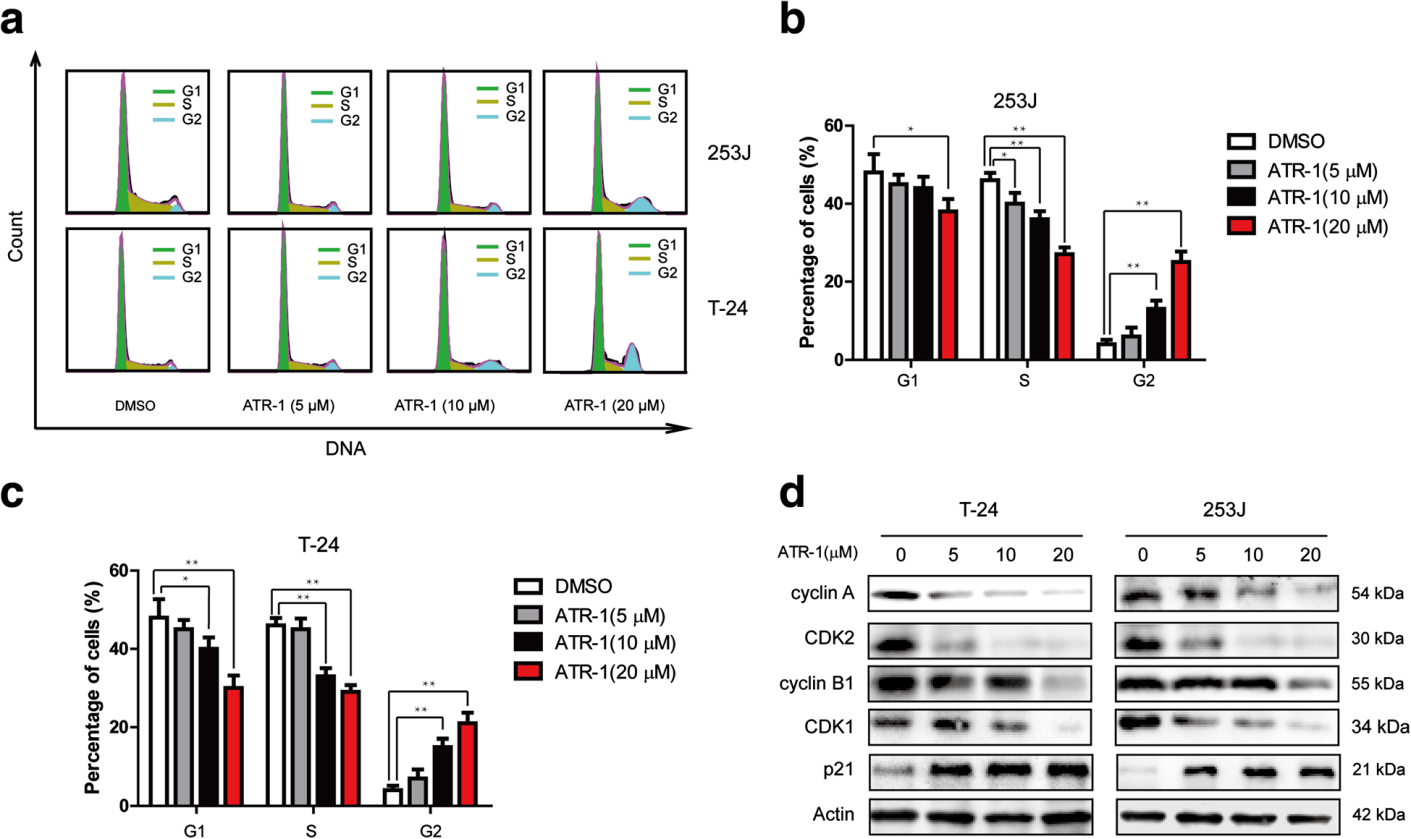
ATR-1 suppressed cell cycle progression in bladder cancer cells. **a** Representative flow cytometry profiles of cell cycle distribution after treatment with DMSO (0.1 %) or various concentrations of ATR-1 for 24 h. **b & c** Quantitative analysis of cell cycle distribution of 253J (b), and T-24 (c) cells treated with DMSO (0.1 %) or indicated concentrations of ATR-1. **d** 253J (right) and T-24 (left) cells were treated with various concentrations of ATR-1 for 24 h, whole cell lysates were analyzed by Western blotting, β-Actin was used as a loading control. Data represent mean ± S.D. (*n* =3).*p < 0.05, **p < 0.01

Anti-tumor chemicals can often inhibit cell proliferation through induction of cell cycle arrest. In order to examine whether the inhibition caused by ATR-1 was a result of cell cycle arrest, the DNA-based cell cycle was analyzed by flow cytometry. Firstly, we incubated cells with various concentrations of ATR-1 for 24 h and then examined the DNA content using propidium iodide (PI) staining. We found that the cell population of ATR-1-treated T-24 and 253J cells was dose-dependently increased in the G2/M and decreased in the S phase, when compared with the control cells which were treated with DMSO (0.1 %) (Fig. 2a, b, c). These results indicate that the inhibitory effect of ATR-1 on T-24 and 253J bladder cancer cell proliferation through accumulating G2/M phase population. Moreover, we tested the effect of ATR-1 on cell cycle checkpoint molecules including cyclin A, cyclin B1, CDK1,CDK2 and p21 by Western blotting. Treatment of cells with ATR-1 dose-dependently increased the expression of p21 while down-regulated cyclin A, cyclin B1, CDK1 and CDK2 in both cell lines (Fig. 2d). These results suggest that the G2/M cell cycle arrest induced by ATR-1 may be related with up-regulation of the cell cycle inhibitory proteins and down-regulation of the cell cycle transition-promoting proteins.

### ATR-1 treatment induced apoptosis in bladder cancer cells

**Fig. 3:**
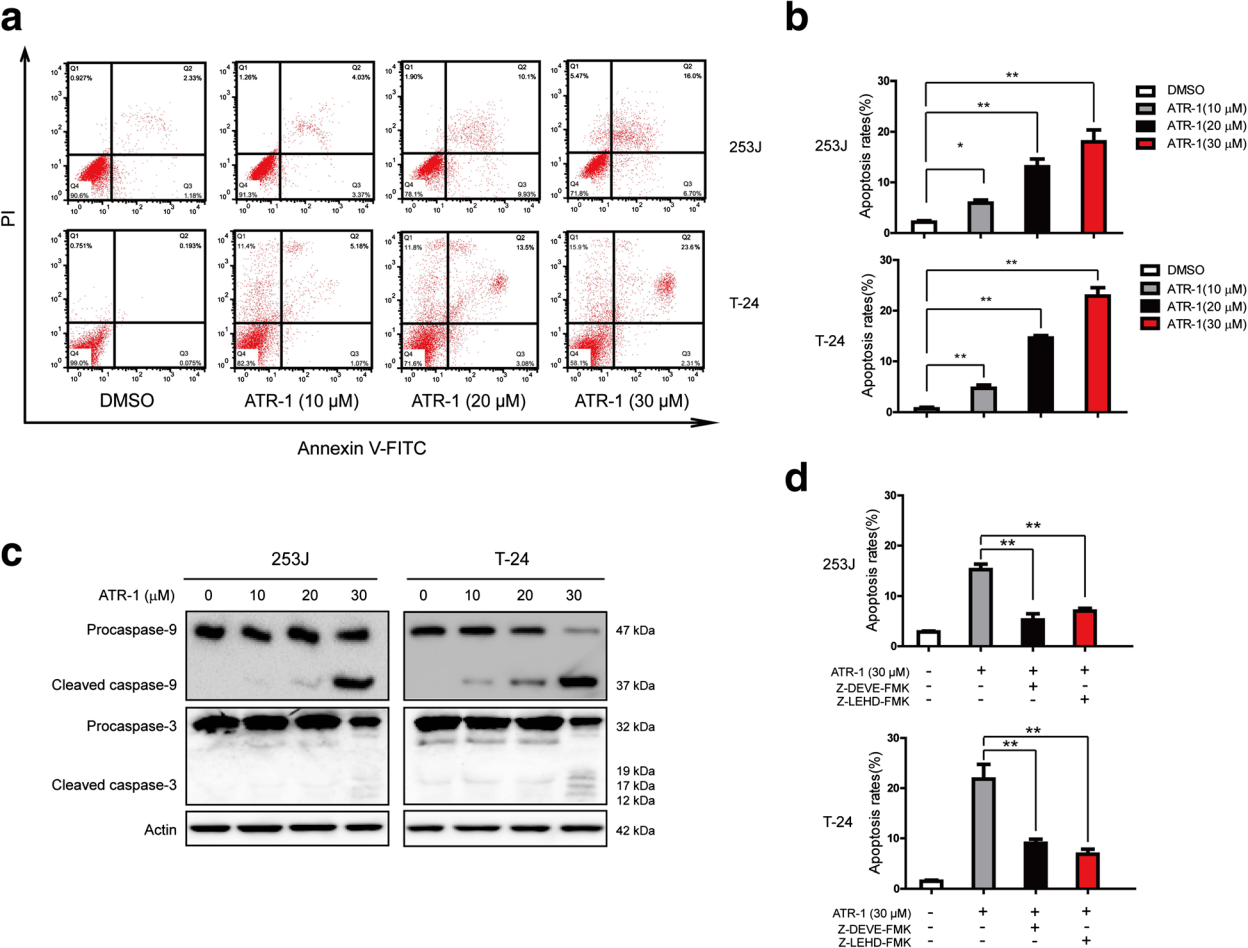
ATR-1 induces apoptosis in bladder cancer cells. **a** Representative flow cytometry profiles of apoptosis induced by different concentrations of ATR-1 in 253J (upper) and T-24 (lower) cells. **b** Quantitative results obtained using Annexin V/PI staining. **c** Western blot analysis of apoptosis-related caspases proteins, β-Actin was used as an equal loading control. **d** Pretreatment of Z-LEHD-FMK, and Z-DEVE-FMK inhibits the apoptosis triggered by ATR-1. Data represent mean ± S.D. (*n* =3).*p < 0.05, **p < 0.01

To further study the anti-tumor activity of ATR-1, we tested whether ATR-1 induces apoptosis in 253J and T-24 cells. We firstly treated cells with ATR-1 at various concentrations for 24 h, and then stained the cells with Annexin V-FITC/PI and measured the proportion of Annexin V/PI positive cells by flow cytometry. As shown in the Fig. 3a and b, ATR-1 was able to induce apoptosis in a dose-dependent manner in both the 253J and T-24 cells. We also performed Western blot to detect caspases activation, results showed that cleaved fragments of caspase-9 as well as caspase-3 were found (Fig. 3c). To gain insight into the role of caspases in the process of ATR-1 induced apoptosis, 253J and T-24 cells were pretreated with specific caspase inhibitors for caspase-9 and caspase-3 (Z-LEHD-FMK and Z-DEVE-FMK, 20 μM, respectively) for 1 h and then incubated with ATR-1 for 24 h. The apoptosis assay revealed significantly reduced apoptosis in the cells pretreated with caspase-9 and caspase-3 inhibitor (Fig. 3d). These results indicate that ATR-1 may induce apoptosis through the intrinsic pathway.

### ATR-1 induces cell apoptosis through mitochondrial pathway

**Fig. 4:**
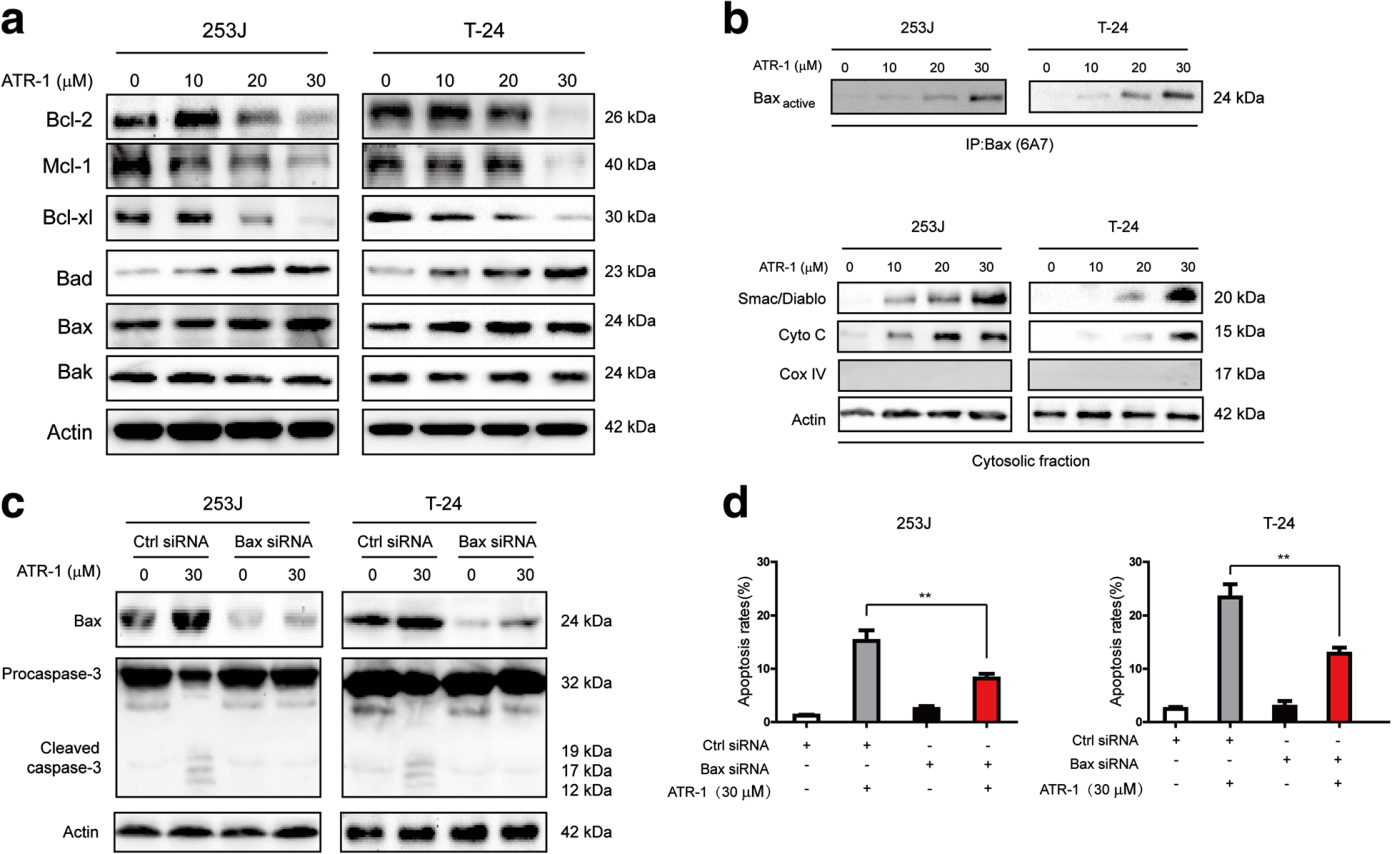
ATR-1 induces apoptosis mainly through the mitochondrial pathway. **a** Western blot analysis of apoptosis-related proteins. **b** ATR-1 treatment can lead to activation of Bax (upper) and release of cytochrome-c (cyto c) and Smac/Diablo into cytosol (lower). **c** Silencing of Bax by specific siRNA can inhibit the activation of caspase-3. **d** Down-regulation of Bax significantly attenuates ATR-1-induced apoptosis. Data represent mean ± S.D. (*n* =3).*p < 0.05, **p < 0.01

In order to further test whether ATR-1 induces apoptosis through the activation of mitochondrial apoptotic pathway, we detected several proteins involved in this pathway. After the treatment of various concentrations of ATR-1 on bladder cancer cells for 24 h, we measured the levels of Bcl-2 family members. As shown in Fig. 4a, the expression levels of anti-apoptotic proteins Bcl-2, Mcl-1 and Bcl-xl were significantly decreased, but pro-apoptotic proteins Bad and Bax were increased while Bak was not affected. In addition, ATR-1 can cause Bax activation (Fig. 4b, upper) and release of cytochrome c and Smac/Diablo into cytosol (Fig. 4b, lower). Moreover, we used siRNA to silence Bax in T-24 and 253J cells (Fig. 4c). The apoptosis induced by ATR-1 and cleavage of caspase-3 were significantly attenuated by silencing of Bax in both cells when compared with control cells that were transfected with scramble siRNA (Fig. 4c and d). Taken together, these results confirm that ATR-1 induces apoptosis in human bladder cancer cells through mitochondrial apoptotic pathway.

### ATR-1 treatment down-regulated PI3K-Akt-mTOR signaling pathway *in vitro*

**Fig. 5:**
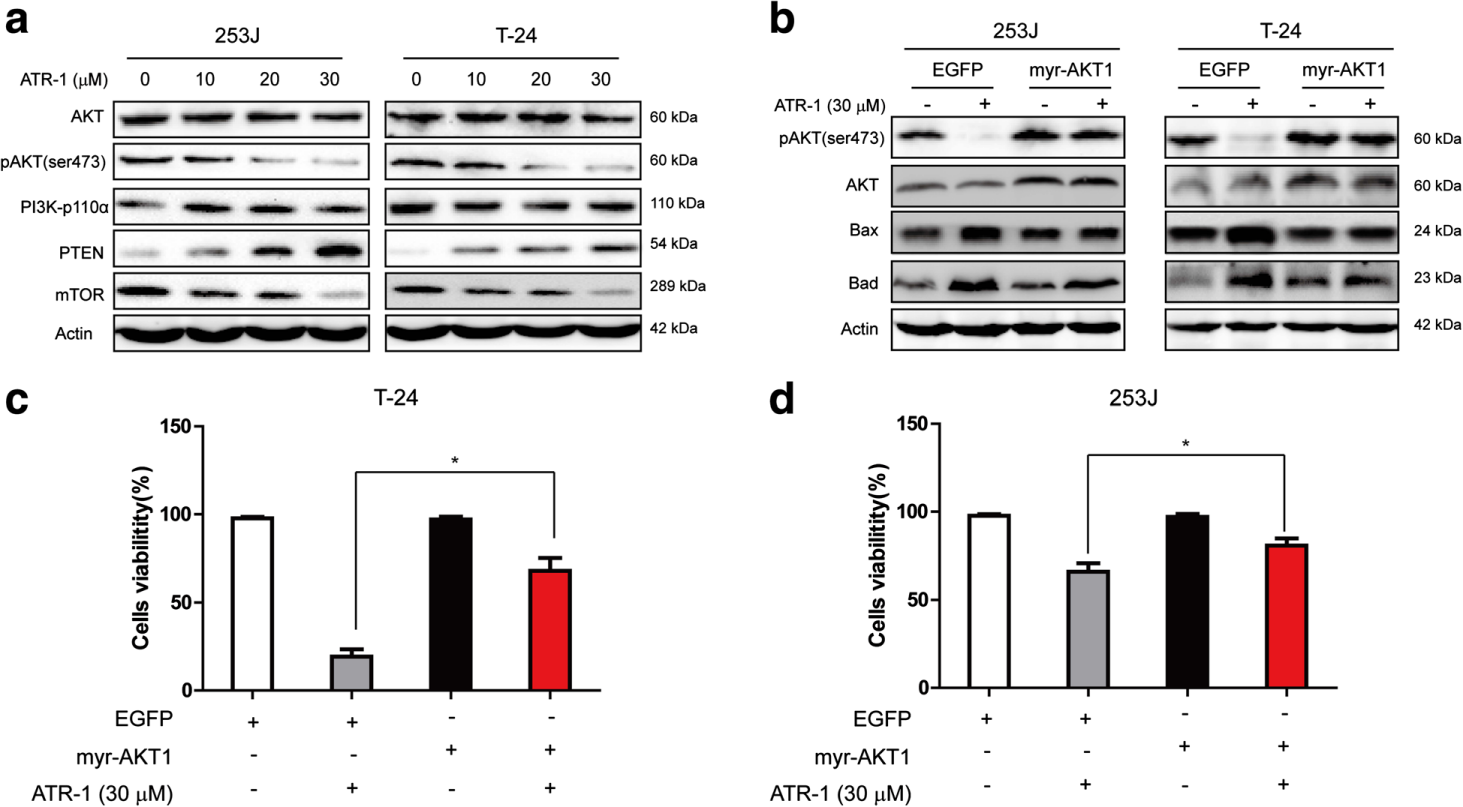
ATR-1 suppressed PI3K/AKT/mTOR signaling pathway. **a** Suppression of phosphorylated AKTSer473, mTOR and induction of PTEN by ATR-1 in 253J (left) and T-24 (right) cells. β-Actin was used as an equal loading control. **b** Analysis of cells transfected with AKT1 constitutively active plasmid or EGFP plasmid (control). Constitutively active AKT1 inhibited the ATR-1 induced up-regulation of Bax and Bad. **c & d** Introduction of constitutively active AKT1 significantly blocks the anti-proliferation activity of ATR-1. Data represent mean ± S.D. (*n* =3).*p < 0.05, **p < 0.01

It is well documented that constitutive activation of PI3K-Akt-mTOR signaling pathway, which contributes to the development of bladder cancer, can also lead to the down-regulation of Bad [17, 20, 21]. Since ATR-1 treatment results in the up-regulation of Bad (Fig. 4a), we hypothesized that PI3K-Akt-mTOR may be involved in the ATR-1 mediated anti-tumor effects. As shown in Fig. 5a, ATR-1 significantly inhibits the phosphorylation of AKT at Ser473 as well as the expression of mTOR, a known downstream target of AKT, in a concentration dependent manner (Fig. 5a). At the same time, there were no changes of the expression of main subunit of PI3K like p110-α and total AKT (Fig. 5a). Moreover, treatment of 253J and T-24 cells with ATR-1 resulted in an increased expression of PTEN, a key negative regulator of PI3K pathway (Fig. 5a). In order to further elucidate the role of PI3K-Akt-mTOR pathway, we transfected 253J and T-24 cells with a plasmid encoding constitutively active AKT1 (myr-AKT1) or EGFP plasmid, 12 h after the transfection we treated the cells with ATR-1 for another 48 h and measured the cells viability. As shown in Fig. 5b-d, over-expression of myr-AKT1 could noticeably rescued the anti-proliferative effect of ATR-1. At the same time, we also measured the levels of Bax and Bad in cells overexpressed of myr-AKT1 to investigate the association between AKT activation and these pro-apoptotic proteins. The western blotting results showed that activation of AKT led to reduction of ATR-1-induced up-regulation of Bax and Bad compared with EGFP-transfected control cells (Fig. 5b). In summary, these data suggest that ATR-1 exerts its anti-tumor function, at least partially, through interfere with the PI3K/AKT/mTOR signaling pathway, which may result in cellular apoptosis in the end.

### Anti-tumor effects of ATR-1 *in vivo*

**Fig. 6:**
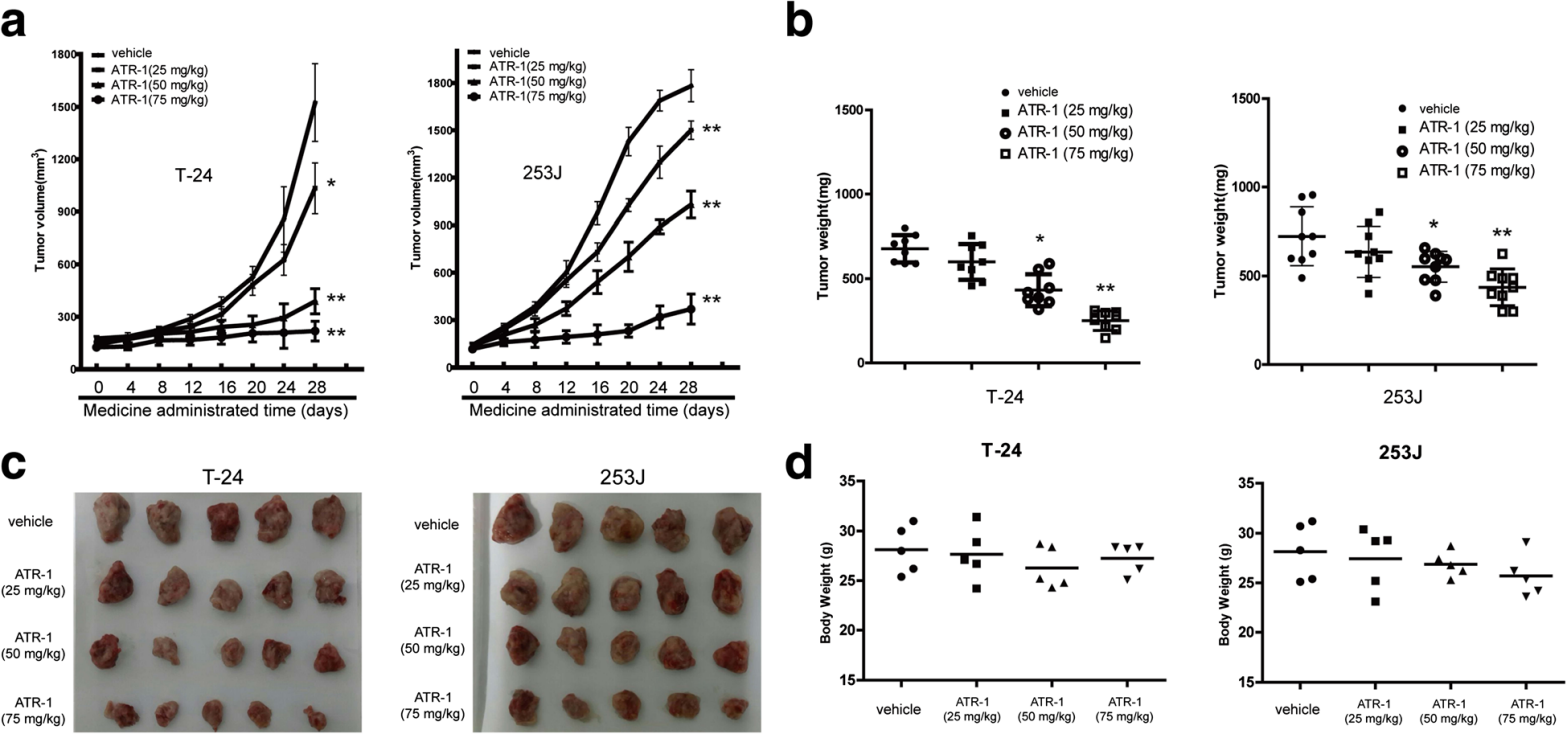
ATR-1 suppressed the growth of bladder cancer in nude mice. **a** Tumor volume changes in T-24 (left) and 253J (right) group after ATR-1 treatment. **b** Tumor weight changes in T-24 (left) and 253J (right) group after ATR-1 treatment. **c** Tumor masses of each group. **d** Body weight changes of each group. Data represent mean ± S.D. (*n* = 6–8). *p < 0.05, **p < 0.01 compared to model group

We then assessed anti-tumor effects of ATR-1 in mouse model. Xenograft tumors were induced by subcutaneous injections of T-24 cells or 253J cells in female BALB/C nude mice. After solid tumor size reached to 100 mm^3^, mice were randomly divided into four groups and administrated with either DMSO (0.1 %) or 25 mg/kg, 50 mg/kg, and 75 mg/kg of ATR-1 once every four days. As shown in Fig. 6a-c, when compared with mice treated with vehicle, mice treated with ATR-1 appeared with reduction of tumor volume as well as tumor weight *in vivo*. Notably, there were no any significant difference of body weights between vehicle treated and ATR-1 treated mice, indicating that ATR-1 can be well tolerant. Together, the results demonstrate that ATR-1 inhibits both xenografted T24 and 253J cells *in vivo*.

## Discussion

ATR-1, an active constituent of *Rhizoma Atractylodis Macrocephalae,* has been shown to have anti-tumor activity though its potential mechanisms are still elusive. In the present study, we found ATR-1 exhibited dose-dependent anti-tumor effects on human bladder cancer cells such as causing cell cycle arrest, inducing apoptosis and inhibition of PI3K/Akt/mTOR signaling pathway.

Unregulated cell cycle is often considered as one of the major characteristics in cancers. Anti-tumor agents often exert their activities through induction of cell cycle arrest. It was well recognized that many cancer cells depend on G2 checkpoint more than normal cells due to defective G1 checkpoint during cell replication [19, 22]. Cell cycle progression is regulated by cyclin-dependent kinases (CDKs) and its regulatory cyclins. The formation of complex CDK1/cyclin B1 is important for the G2/M phase transition of cell cycle. Activation of CDK1 requires the specific phosphatase Cdc25, which dephosphorylate CDK1 at positions Thr14 and Tyr15, thereby permitting cell entry into the M phase [23]. In our study, we found that treatment of ATR-1 leads to the G2/M arrest in 253J and T-24 cells (Fig. 2a, b, c), which is accompanied by the down-regulation of cyclin A, cyclin B1, CDK1 and CDK2 whereas increased the level of p21 (Fig. 2d). In a recent paper, Ye et al. reported that ATR-1 induced cell cycle arrest at G1 phase in melanoma cells [10]. The discrepancy may be caused by difference of cell types, and the exact mechanism require further investigation.

Plenty of studies have proven that the activation of the apoptotic pathway in cancer cells is a defensive mechanism against the progression and development of tumor. In the past years, many compounds have been found to promote apoptosis by triggering mitochondrial pathway, such as isoorientin, arsenic trioxide and quinazolinone-chalcone derivative [24–26]. In the mitochondrial apoptotic pathway, mitochondrial membrane permeabilization is tightly regulated by the interaction of pro- and anti- apoptotic members of Bcl-2 family [27]. The ratio between anti- and pro-apoptotic Bcl-2 family members has been known as a determination whether a cell will undergoes apoptosis [27]. The mitochondrial permeability can be enhanced by the activation of Bax and formation of channels in the membrane, which resulted in the release of cytochrome c and Smac/Diablo [27]. Release of cytochrome c and Smac/Diablo then led to the activation of caspase-9, caspase-3 and finally apoptosis. Our results showed that treatment of bladder cancer cells 253J and T-24 led to the increase of cleaved caspase-9 and caspase-3 (Fig. 3c). We also detected the release of cytochrome c, Smac/Diablo into cytosol and activation of Bax (Fig. 4b). Meanwhile, the levels of pro-apoptotic Bcl-2 members Bax, Bad were increased and anti-apoptotic Bcl-2, Bcl-xl, Mcl-1were decreased (Fig. 4a). The use of caspase-9, caspase-3 inhibitors significantly blocked the ATR-1 induced apoptosis (Fig. 3d). Similarly, silencing of Bax by specific siRNA also inhibited the apoptosis as well as the caspase-3 activation (Fig. 4c, d). This implicats that Bax was critically involved in ATR-1 induced apoptosis and highlights the link between PI3K/Akt/mTOR and Bax. Taken together, these data indicate that ATR-1-induced apoptosis in human bladder cancer cells is dependent on activation of mitochondrial apoptotic pathway.

Currently, there are three known classes of PI3 kinases including classes I, II and III, which are classified by structure and function. The class I PI3K, which has four isoforms (PI3Kα, PI3Kβ, PI3Kδ and PI3Kγ), is the most studied isoform in human cancers [28]. AKT, a downstream effector of PI3K, has three isoforms: Akt1, Akt2 and Akt3. mTOR is another downstream effector of PI3K and involved in several cellular activities such as protein synthesis, translation initiation, cell mass, etc [15, 29]. PI3K/Akt/mTOR pathway is vital for cancer cell survival, motility, metabolism, migration and drug resistance. It is estimated that approximately 40 % of urothelial carcinomas have PI3K/Akt/mTOR pathway activating mutations [1, 30]. Activation of PI3K/Akt/mTOR also contribute to the migration, invasion and chemoresistance of bladder cancer [31, 32]. In our study, western blotting analysis indicates that phosphorylation of Akt at Ser473 position was decreased by ATR-1 treatment in both bladder cancer cell lines *in vitro* (Fig. 5a). Meanwhile, transfection of cells with constitutively active Akt1 greatly blocked the inhibitory effect of ATR-1 (Fig. 5c, d) as well as the up-regulation of Bax and Bad (Fig. 5b), thereby proving that the anti-tumor effect of ATR-1 relies on the inhibition of PI3K/Akt/mTOR pathway. In addition, we also observed increase of expression of PTEN after ATR-1 treatment (Fig. 5a). PTEN is a lipid phosphatase that directly antagonises the activity of PI3K [28]. Loss or down-regulation of PTEN represents one of the most common alterations in bladder carcinoma [17]. These data indicated that the PI3K/Akt/mTOR pathway was involved in apoptosis induced by ATR-1.

To our knowledge, ATR-1 has several merits that may facilitate its usage in the future. First, clinic evidences suggest that ATR-1 is safe and low toxic without serious adverse reactions after taken by patients [12]. Second, a recent work reported ATR-1 sensitized human ovarian cancer cells to paclitaxel, which indicates the combination of ATR-1 with current chemotherapeutics may overcome drug resistance [33]. Third, active components in RAM were supposed to have immune-enhance activity as one recent study found extracts from RAM significantly enhanced T lymphocyte proliferation individually or synergistically [34]. Thus, ATR-1 is a valuable natural product which may play a role in the treatment of bladder cancer individually or in combination with other chemotherapeutic reagents. Further studies need to be carried out to investigate ATR-1 therapeutic value in bladder cancer.

## Conclusions

In summary, we have tested the potential therapeutic function of ATR-1 on two human bladder cancer cell lines *in vitro* and *in vivo*. ATR-1suppresses xenograft tumor growth, triggers G2/M cell cycle arrest, induces apoptosis through intrinsic pathway and blocks PI3K/Akt/mTOR signaling pathway. Thereby, ATR-1 may be developed as an effective agent to treat human bladder cancer by further investigation.

## Abbreviations

ATR-1: Atractylenolide I
CDKs: cyclin-dependent kinases
MMP: mitochondrial membrane potential
mTOR: the mammalian target of rapamycin
MTT: Methyl thiazolyl diphenyl tetrazolium bromide
PARP-1: poly(ADP-ribose) polymerase-1
PI: propidium iodide
PI3K: phosphatidylinositol 3-kinase
PTEN: phosphatase and tensin homolog deleted on chromosome ten
RAM: *Rhizoma Atractylodis Macrocephalae*
