# Correction to: Anti-tumor effects of Atractylenolide I on bladder cancer cells

**DOI:** 10.1186/s13046-018-0913-1

**Published:** 2018-10-15

**Authors:** Rui Yu, Bi-xia Yu, Jun-feng Chen, Xiu-yi Lv, Ze-jun Yan, Yue Cheng, Qi Ma

**Affiliations:** 1 Department of Biochemistry and Molecular Biology, Zhejiang Key Laboratory of Pathophysiology, School of Medicine, Ningbo University, Fenghua St, Ningbo, 315211 China; 2 Translational Research Laboratory for Urology, Ningbo First Hospital, The Affiliated Hospital of Ningbo University, Liuting St, Ningbo, 315010 China; 3 Department of Urology, Ningbo First Hospital, The Affiliated Hospital of Ningbo University, Liuting St, Ningbo, 315010 China

## Correction

In the publication of this article [1], there is an error in Fig. 4b. The Cyto c western blot image in Fig. 4b is misrepresented. This has now been included in this correction. The authors declare that the correction does not change the results or conclusions of this paper.

The error Fig. 4b given hereafter:

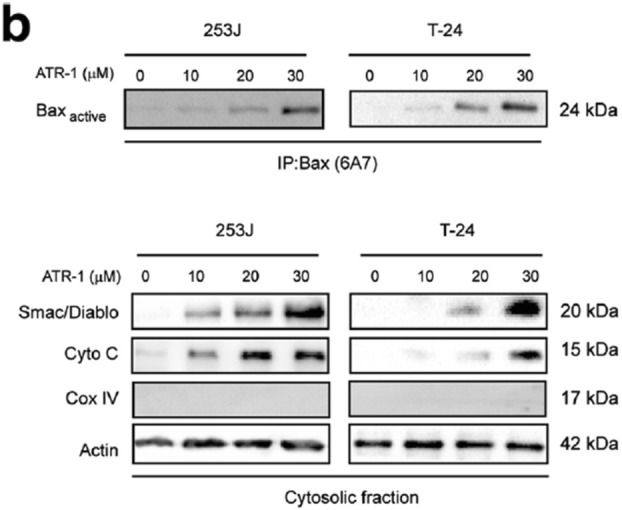



The correct Fig. 4b given hereafter:


